# Attenuation of multiple Nef functions in HIV-1 elite controllers

**DOI:** 10.1186/1742-4690-10-1

**Published:** 2013-01-07

**Authors:** Philip Mwimanzi, Tristan J Markle, Eric Martin, Yoko Ogata, Xiaomei T Kuang, Michiyo Tokunaga, Macdonald Mahiti, Florencia Pereyra, Toshiyuki Miura, Bruce D Walker, Zabrina L Brumme, Mark A Brockman, Takamasa Ueno

**Affiliations:** 1 Center for AIDS Research, Kumamoto University, 2-2-1 Honjo, Kumamoto, 860-0811, Japan; 2 Simon Fraser University, Burnaby, BC, Canada; 3 Ragon Institute of Massachusetts General Hospital, Massachusetts Institute of Technology and Harvard University, Boston, MA, USA; 4 University of Tokyo, Tokyo, Japan; 5 British Columbia Centre for Excellence in HIV/AIDS, Vancouver, BC, Canada

**Keywords:** HIV-1, Nef, Elite controllers, Human Leukocyte Antigen (HLA) class I, Immune escape, Replication capacity, HLA-B*57

## Abstract

**Background:**

Impaired HIV-1 Gag, Pol, and Env function has been described in elite controllers (EC) who spontaneously suppress plasma viremia to < 50 RNA copies/mL; however, activity of the accessory protein Nef remains incompletely characterized. We examined the ability of 91 Nef clones, isolated from plasma of 45 EC and 46 chronic progressors (CP), to down-regulate HLA class I and CD4, up-regulate HLA class II invariant chain (CD74), enhance viral infectivity, and stimulate viral replication in PBMC.

**Results:**

In general, EC Nef clones were functional; however, all five activities were significantly lower in EC compared to CP. Nef clones from HLA-B*57-expressing EC exhibited poorer CD4 down-regulation function compared to those from non-B*57 EC, and the number of EC-specific B*57-associated Nef polymorphisms correlated inversely with 4 of 5 Nef functions in these individuals.

**Conclusion:**

Results indicate that decreased HIV-1 Nef function, due in part to host immune selection pressures, may be a hallmark of the EC phenotype.

## Background

Elite controllers (EC) are rare (<1%) HIV-1 infected individuals who spontaneously suppress plasma viral loads to undetectable levels in the absence of antiviral therapy. Several factors likely contribute to this phenotype, including host genetics [1], characteristics of HLA-restricted T-cell responses [2], immune-mediated reductions in viral protein function and/or replication [3,4], and acquisition of attenuated viruses [5,6]. Recombinant viruses expressing *gag* and *pol* sequences from EC exhibit reduced *in vitro* replication capacity, due in part to cytotoxic T lymphocyte (CTL) escape mutations selected by certain HLA class I (HLA-I) alleles [3,4], while EC-derived viral envelopes exhibit impaired entry [7]. The *in vitro* function of other viral proteins in EC remains incompletely characterized.

HIV-1 Nef is an accessory protein required for maintenance of high viral loads and progression to AIDS [8], as demonstrated by slow or non-progressive disease in hosts infected with *nef*-deleted or otherwise *nef*-defective strains [5,6,9,10]. Nef exhibits a variety of *in vitro* functions that may modulate pathogenesis, including CD4 down-regulation [11], HLA-I down-regulation [12], HLA class II invariant chain (CD74) up-regulation [13], enhancement of virion infectivity [14], and stimulation of viral replication in PBMC [15] (for reviews see [16-18]). Multiple Nef activities may act together to facilitate immune evasion and enhancement of viral spread *in vivo*[19]; however, multi-functional assessments of patient-derived Nef clones from HIV elite controllers are lacking. Although Nef sequence diversity is highly influenced by host HLA-I selection pressures [20], the relationship between HLA-associated polymorphisms and Nef function is largely unknown. Assessing multiple *in vitro* Nef functions in EC, a population that is highly enriched for protective HLA-I alleles such as B*57 [1], provides an opportunity to investigate these questions.

Previous analysis of plasma HIV RNA Nef sequences in our cohort of EC revealed no evidence of gross mutational defects [21], suggesting that any impairment in Nef protein function would have a more complex etiology. For this study, we generated recombinant viruses encoding a single representative HIV RNA Nef clone from 45 EC to assess Nef-mediated down-regulation of HLA class I, up-regulation of HLA class II invariant chain (CD74), viral infectivity, and viral replication in PBMC. The same Nef clone was engineered into a GFP-expression vector to assess its ability to down-regulate CD4. Results were compared to the activities of HIV RNA-derived Nef clones from 46 chronic progressors (CP). Finally, we assessed the role of host immune selection pressures, most notably novel polymorphisms associated with HLA-B*57 in EC, on Nef function in these individuals.

## Results

### Nef protein expression and viral production

**Figure 1:**
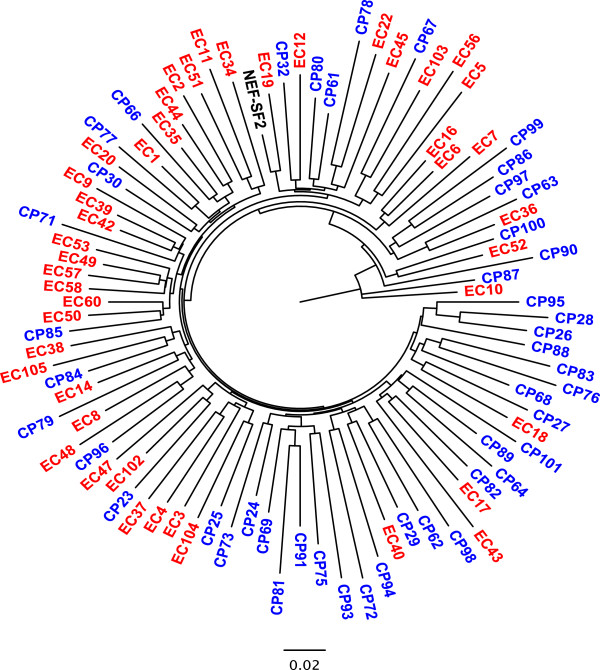
**Maximum-likelihood phylogenetic tree of plasma HIV RNA-derived Nef clonal sequences.** EC-derived Nefs are red, CP-derived Nefs are blue, and control strain SF2 is black.

**Figure 2:**
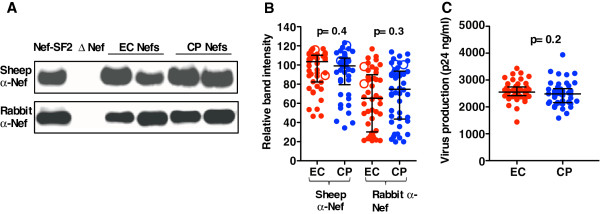
**Nef expression and progeny virus production. ***Panel ***A**: Western blot detection of control strains NL4.3-Nef_SF2_ and NL4.3-ΔNef and two representative EC and CP-derived Nefs using two different polyclonal anti-Nef sera. *Panel ***B**: Relative band intensity of EC (red) and CP (blue)-derived Nefs as detected by Western blot. Open circles identify the representative data shown in panel A. Bars depict median and interquartile ranges. Statistical significance assessed using Mann–Whitney U-Test. *Panel ***C**: Virus production (measured as p24^Gag^ in culture supernatant) of EC (red) and CP (blue) Nef recombinant viruses.

For each of 45 EC and 46 CP, a single representative plasma HIV RNA-derived Nef sequence with an intact open reading frame (ORF) was cloned into a recombinant NL4.3 virus construct. Consistent with previous analyses of bulk plasma HIV RNA sequences from our EC cohort [21], clonal Nef sequences from EC showed no evidence of gross defects or recent shared ancestry (Figure 1, Additional file 1: Table S1). Western blots revealed comparable band intensities between EC and CP, indicating that EC Nefs were not markedly diminished in steady-state protein expression levels (Figure 2A, B). Similarly, p24^Gag^ levels in culture supernatants were comparable between groups, indicating that EC Nefs were not significantly impaired in virion production (Figure 2C).

### Nef-mediated enhancement of viral infectivity and replication is impaired in EC

**Figure 3:**
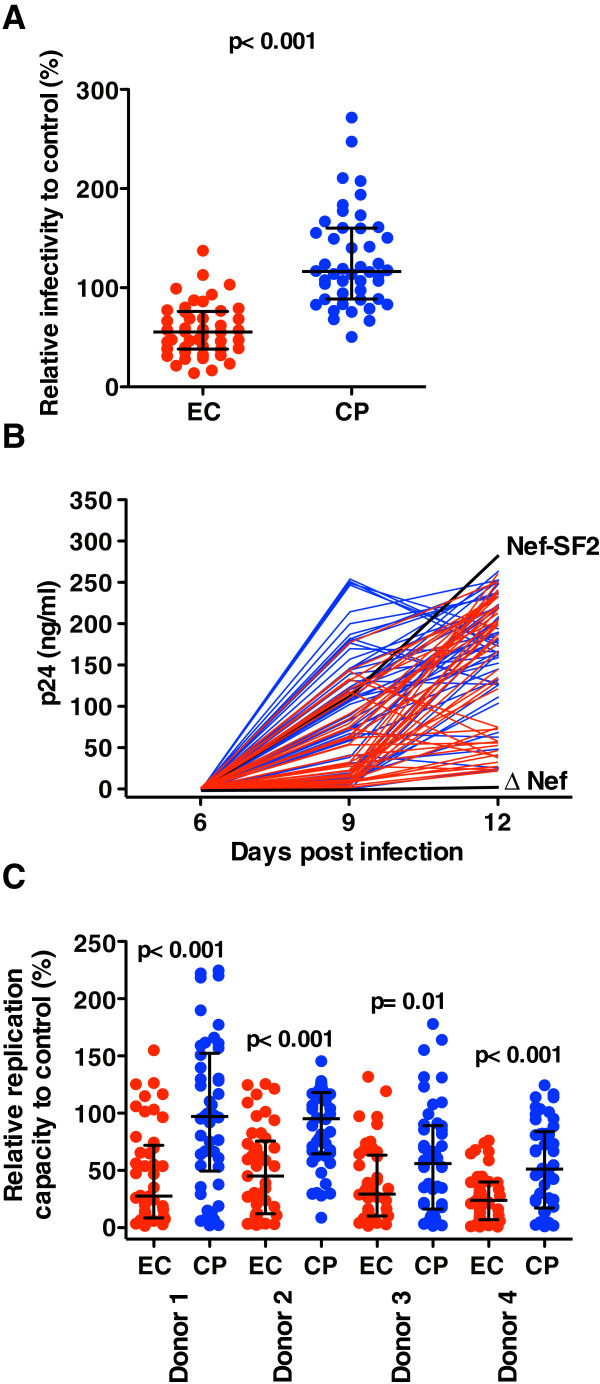
**Ability of EC Nef to enhance viral infectivity and stimulate viral replication in PBMC. ***Panel ***A**: Scatterplots depict the ability of EC (red) and CP (blue) Nef recombinant viruses to enhance viral infectivity. Values are normalized to that of NL4.3-Nef_SF2_ such that values of 100% indicate infectivity equal to that of NL4.3-Nef_SF2_, while values <100% and >100% indicate infectivity lower than or higher than that of NL4.3-Nef_SF2_, respectively. Bars represent median and interquartile ranges. The p-value was calculated using Mann–Whitney U-test. *Panel ***B**: Growth curves are shown for EC (red) and CP (blue) Nef recombinant viruses, plus control viruses NL4.3-Nef_SF2_ and NL4.3-ΔNef, in PBMCs from donor #1. The mean value from quadruplicate samples is shown at each time point. The means ± SEM values for control viruses NL4.3-Nef_SF2_ and NL4.3-ΔNef at day 9 and 12 are 115 ± 27 and 1.3 ± 0.02, and 285 ± 18.8 and 4.3 ± 0.4 respectively. *Panel ***C**: Scatterplots depict the ability of recombinant EC (red) and CP (blue) Nef recombinant viruses to stimulate viral replication in PBMC from four HIV-negative donors. Values are normalized to that of control NL4.3-Nef_SF2_. Bars represent median and interquartile ranges. P-values were calculated using Mann–Whitney U-test.

All viruses harboring EC Nef displayed infectivity greater than the negative control NL4.3ΔNef, which had 7.3% infectivity relative to NL4.3-Nef_SF2_. Compared to control strain NL4.3-Nef_SF2_, median EC Nef infectivity was 55% (IQR 38-76%), values that were significantly lower than CP-derived Nef (median 116, IQR 88-160%) (p < 0.001; Figure 3A). Similarly, all viruses harboring EC Nef displayed higher replication capacities than the negative control NL4.3ΔNef in PBMCs from four HIV-negative donors. Consistent with previous reports [22,23], replication of patient-derived Nef recombinant viruses in PBMC differed to some extent among donors; however, viruses encoding EC Nef displayed consistently poorer ability to replicate in PBMC relative to those harboring CP Nef in all donors (p ≤ 0.01; Figure 3B, C). Averaged over all four donors, median [IQR] replication capacities were 34 [23–52]% of NL4.3-Nef_SF2_ for EC-derived viruses and 76 [57–98]% for CP-derived viruses, respectively (p < 0.001, not shown).

### Modulation of surface HLA-I, CD74, and CD4 by EC Nef

**Figure 4:**
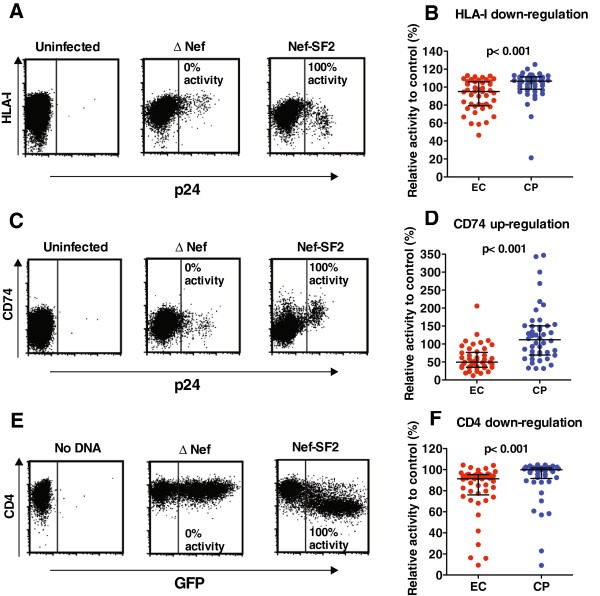
**Ability of EC Nef to modulate cell surface receptor levels. ***Panel ***A**: Flow cytometry plots depicting representative staining of cell-surface HLA-I (HLA-A*2402; y-axis) vs. intracellular p24^Gag^ (x-axis) for uninfected, NL4.3-ΔNef (negative) and NL4.3-Nef_SF2_ (positive) controls are shown. *Panel ***B**: Scatterplots depicting the ability of recombinant EC (red) and CP (blue) Nef recombinant viruses to down-regulate HLA-I are shown. *Panel ***C**: Flow cytometry plots depicting representative staining of cell-surface CD74 (y-axis) vs. intracellular p24^Gag^ (x-axis) using uninfected and control viruses are shown. *Panel ***D**: Scatterplots depicting the ability of recombinant EC (red) and CP (blue) Nef recombinant viruses to upregulate CD74 are shown. *Panel ***E**: Flow cytometry plots depicting representative staining of cell-surface CD4 (y-axis) vs. GFP (x-axis) after delivery of no-DNA, ΔNef and Nef_SF2_ plasmid vectors are shown. *Panel ***F**: Scatterplots depicting the ability of EC (red) and CP (blue)-derived Nef to downregulate CD4 are shown. In these experiments, results are normalized to NL4.3-Nef_SF2._ (positive) and NL4.3-ΔNef (negative) controls. The activity of NL4.3-ΔNef or ΔNef plasmid is inherently set to zero. Bars represent median and interquartile ranges. P-values were calculated using Mann–Whitney U-test.

All EC Nef clones displayed greater ability to modulate cell-surface receptors than ΔNef negative controls. Relative to control strain NL4.3-Nef_SF2_, EC-derived Nef recombinant viruses maintained considerable HLA-I down-regulation activity (median 95 [IQR 79–106]%) that was nevertheless significantly lower compared to CP Nef viruses (median 106 [IQR 96–111]%) (p<0.001; Figure 4A, B). The ability of EC Nef viruses to up-regulate CD74 was markedly lower (median 49 [IQR 35–76]%) compared to CP Nef viruses (median 111 [IQR 68–150]%) (p < 0.001; Figure 4C, D). HIV-1 Vpu and Env proteins contribute to surface CD4 modulation [24]; therefore, Nef-mediated CD4 down-regulation activity was assessed using DNA expression plasmids. Relative to control Nef_SF2_, most EC Nef clones maintained substantial CD4 down-regulation activity (median 91 [IQR 76–95]%) that was nevertheless significantly lower compared to CP Nef clones (median 99 [IQR 89–101]%) (p = 0.002) (Figure 4E, F). All EC Nef sequences and functional data are provided in Additional file 1: Table S1. Of interest, 32 of 45 (71%) EC Nef viruses displayed replication activity less than 50% of that of control strain Nef_SF2_, while only one EC Nef virus showed HLA-I down-regulation activity less than 50%. Three EC Nef demonstrated activity less than 50% of Nef_SF2_ for all functions tested, except HLA-I down-regulation (Additional file 1: Table S1).

### Host HLA-I allele expression and Nef function in EC

Protective HLA-I alleles, most notably B*57, are over-represented in EC [1,25]. To investigate this as a potential confounder in comparisons between EC and CP, we re-analyzed our data excluding individuals who expressed HLA-B*57 (17/45 of EC and 8/46 of CP). Measures for all five Nef functions remained significantly lower among non-B*57 EC compared to non-B*57 CP (all p < 0.01, not shown). Exclusion of individuals expressing any protective allele (defined as B*27, B*57, and B*58:01) yielded similar results (all p < 0.05, not shown).

**Figure 5:**
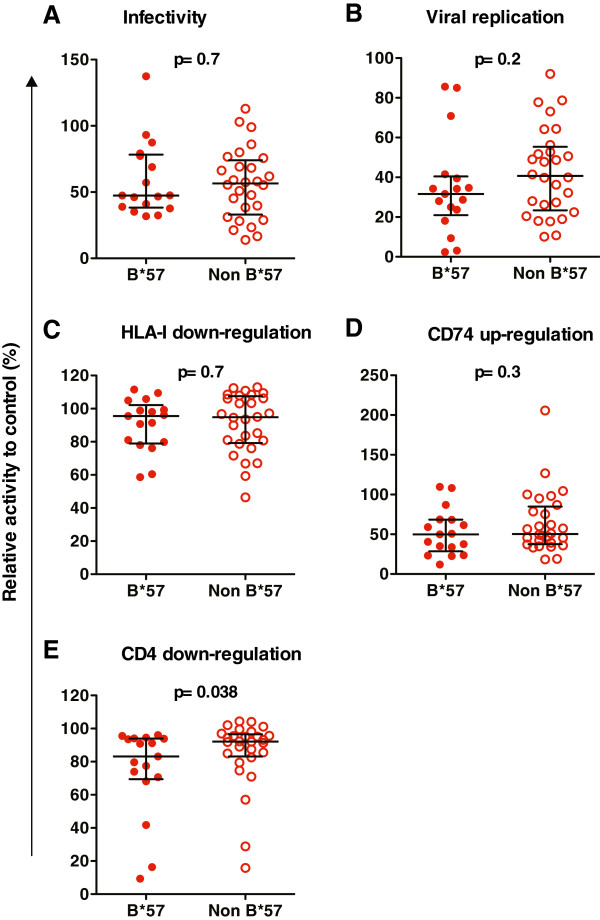
Relationship between host HLA-B*57 expression and Nef activities in EC. Scatterplots are shown depicting the ability of EC-derived Nefs from B*57-expressing (closed red circles) and non-B*57 expressing (open red circles) to enhance virion infectivity (*panel ***A**), enhance viral replication in PBMC (*panel ***B**; data depict means for all four PBMC donors), down-regulate HLA-I (*panel ***C**), up-regulate CD74 (*panel ***D**) and down-regulate CD4 (*panel ***E**). All results are normalized to Nef_SF2_. Bars represent median and interquartile ranges. P-values were calculated using Mann–Whitney U-test.

Immune selection by protective HLA-I alleles, including B*57, can modulate the *in vitro* function of certain HIV-1 proteins in EC [3,4]. To examine whether this was also true for Nef, we stratified EC Nef clones by host B*57 expression and observed significantly lower CD4 down-regulation activity in B*57-derived compared to non-B*57-derived EC Nefs (median [IQR] 83 [55–94]% for B*57 vs. 92 [83–97]% for non-B*57 EC, respectively, p = 0.038). Significant differences were not seen for the other Nef activities tested (Figure 5). Of 20 HLA-I alleles expressed in a minimum of five EC, correlations with Nef function were also observed for C*06 (in linkage disequilibrium with B*57; median 74 vs 93% CD4 down-regulation activity in C*06 vs. non-C*06 EC) and A*01 (median 83 vs 97% HLA-I down-regulation activity in A*01 vs. non-A*01 EC) (both p < 0.05; q < 0.05). No HLA-I associations were observed for Nef-mediated infectivity, replication, or CD74 up-regulation activity in EC.

### Unique HLA-associated polymorphisms and Nef function in EC

**Figure 6:**
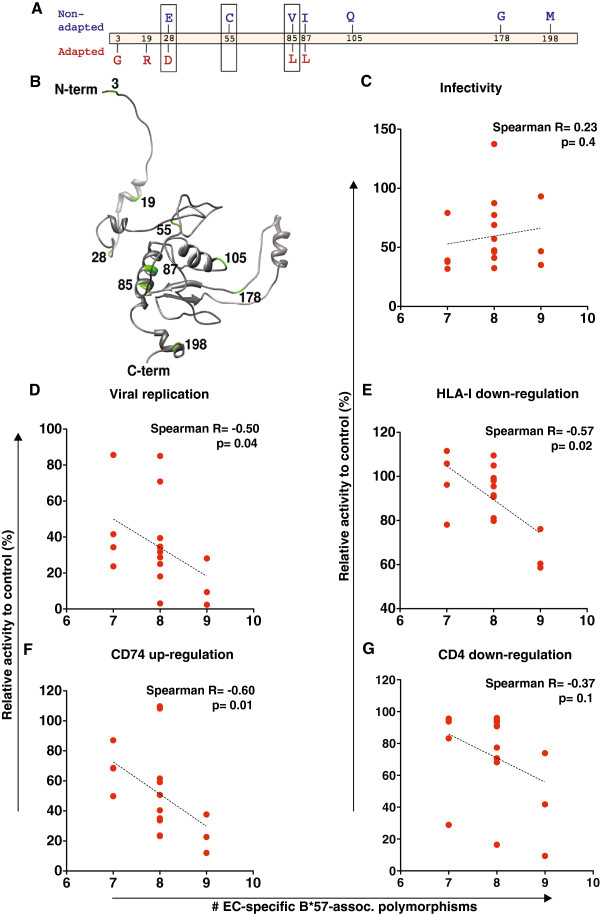
HLA-B*57-associated polymorphisms in EC, and their relationship with Nef function. *Panel ***A**: Two-dimensional map of B*57-associated polymorphisms identified in an exploratory analysis of the EC cohort using phylogenetically-corrected approaches with p<0.05 [20]. “Nonadapted” forms (those less likely to be observed in the presence of B*57 at a specific location) are shown above the Nef protein in blue; “adapted” forms (those enriched among B*57-expressing persons) are shown below the Nef protein in red. Due to limited statistical power and other reasons, both nonadapted and adapted forms are not always identified at a given position. Boxed codons indicate those where the amino acid varied in B*57 EC; the remainder were either expressed in 100% of B*57 EC (adapted forms 3G and 19F) or 0% of B*57 EC (nonadapted forms 105Q, 178G, 198M). In subsequent analyses, Nef sequences from B*57-expressing persons were counted as harboring a B*57-associated polymorphism at a given site if that site expressed anything other than the nonadapted form, or the adapted form if no nonadapted form was identified. *Panel ***B**: The locations of the nine EC-specific B*57-associated polymorphisms are indicated in green on the structure model of the Nef protein (composite crystal structure kindly provided by Art F. Y. Poon, [31]) *Panels ***C-G**: Correlations between the number of B*57-associated polymorphisms in Nef sequences from B*57-expressing EC, and five Nef functions evaluated. Statistical analyses were done using Spearman’s correlation.

Modulation of viral protein function in EC by protective HLA-I alleles may be due to the selection of unconventional HLA-associated polymorphisms in this patient group [26,27]. To examine this, we applied phylogenetically-corrected methods [28] to identify HLA-B*57-associated Nef polymorphisms in our cohort of 45 EC. Nine associations were observed at p < 0.05 (q < 0.4) in B*57^+^ EC (Figure 6A). With the exception of V85L, these B*57-associated polymorphisms were distinct from those previously identified in large population-level analyses of chronically subtype B infected individuals (N >1500) [20,29], suggesting that they may be largely unique to EC. In contrast, a search for B*57-associated polymorphisms in our cohort of 46 CP revealed several expected Nef polymorphisms at p < 0.05, including V85L and H116N [20,29,30] (not shown), supporting our ability to identify HLA-associated polymorphisms in cohorts of the present size. Therefore, we reasoned that the unconventional B*57-associated polymorphisms observed in EC merited further attention.

Among B*57-expressing EC (N = 17), we observed significant inverse relationships between the number of EC-specific B*57-associated polymorphisms and Nef-mediated replication (Spearman R = −0.5, p = 0.04), HLA-I down-regulation (R = −0.57, p = 0.02) and CD74 up-regulation (R = −0.6, p = 0.01); and a modest, albeit not statistically significant, negative correlation with CD4 down-regulation (R = −0.37, p = 0.1) (Figure 6). No similar correlations were observed between EC-specific polymorphisms associated with other HLA-I alleles observed at comparable frequencies in our cohort (i.e. A*01, A*02, A*03, A*30, C*06, C*07) and Nef function in EC expressing these alleles (not shown).

### Amino acids associated with EC Nef function

To investigate the relationship between Nef sequence and function in EC, we performed an exploratory analysis to correlate amino acids with each of the five functions tested, regardless of host HLA. A total of 23 polymorphisms occurring at 14 sites were associated with Nef-mediated modulation of HLA-I, CD74 and CD4 (p < 0.05, q < 0.4; Table 1). No Nef polymorphisms associated with infectivity or replication were observed at this threshold.

**Table 1:** Analysis of Nef residues associated with EC Nef functions (all N>5 and q<0.4)

**Nef activity**	**Position on HXB2**	**AA**^**a**^	**No. of subjects**^**b**^		**Relative Nef activity (%)**		**p-value**	**q-value**
			**With AA**	**Without AA**	**With AA**	**Without AA**		
HLA-I down-regulation	8	S	17	27	83.6	97.2	0.01	0.3
	8	R	14	30	106.1	91.1	0.02	0.3
	11	V	20	21	87.2	98.9	0.01	0.3
	14	P	34	11	91.1	108	0.004	0.3
	46	S	36	8	96.7	77.1	0.01	0.3
	108	D	34	11	91.1	105.8	0.02	0.3
	108	E	11	34	105.8	91.1	0.02	0.3
	138	C	5	40	79.8	96.5	0.02	0.3
	138	T	40	5	96.5	79.8	0.02	0.3
	192	H	36	9	97	78.1	0.01	0.3
	192	R	6	39	84	96.8	0.04	0.4
	205	N	22	23	101	83.6	0.02	0.3
	205	D	22	23	85.9	98.9	0.03	0.3
CD74 up-regulation	8	S	17	27	37.7	60	0.004	0.3
	28	E	14	31	73.7	42.4	0.008	0.3
	28	D	29	16	40.4	65.3	0.009	0.3
	135	F	10	35	35	57.5	0.003	0.3
	135	Y	34	11	57.1	35.1	0.01	0.3
CD4 down-regulation	8	S	17	27	84.8	92.6	0.007	0.3
	14	P	34	11	86.1	97.9	0.003	0.1
	14	S	5	40	102.9	87.2	0.02	0.4
	15	A	11	34	76.9	91.3	0.02	0.4
	21	K	11	34	70.7	91.6	0.002	0.1
	21	R	28	17	91.6	76.9	0.02	0.4
	105	R	12	33	76.6	91.9	0.003	0.1
	163	C	9	36	98.1	86.6	0.02	0.4

^**a**^ AA, amino acid.

^**b**^ Row totals vary depending on the codon position because sequences with gaps in the alignment are considered missing data.

## Discussion

We assessed five *in vitro* Nef functions using clonal plasma HIV RNA sequences from 45 EC and 46 CP. We observed that EC Nef clones were generally functional, especially for Nef’s most characteristic activities, CD4 and HLA-I down-regulation. Nevertheless, median EC Nef activities were significantly lower for all five functions when compared to those from CP. Median CP Nef activities were consistent with that of HIV-1 strain SF2 used as a normalization control for all assays, indicating that our selection of chronic Nef clones is representative of chronic Nef isolates examined previously. The range in Nef activities observed here may help to resolve discrepancies between previous studies of HIV long-term non-progressors or controllers, which have reported relative preservation of CD4 and/or HLA-down-regulation function [32,33], inefficient Nef-mediated CD4 and/or HLA-down-regulation [34-36] and reduced infectivity [35] compared to CP. Our data suggest that there is *in vivo* pressure on Nef in EC to maintain CD4 and HLA-I down-regulation functions.

Relative functional impairments between EC and CP clones are not likely to be due to differences in Nef protein stability or expression levels, nor to recent descent from a defective common ancestor. Similarly, while enrichment of protective HLA alleles in EC may contribute to Nef sequence, it is not likely to be the only explanation for relative functional attenuation observed here, since differences between groups persisted after persons who expressed protective HLA alleles were excluded from analysis. Indeed, although significantly lower CD4 down-regulation activity was observed in B*57 compared to non-B*57 EC (Figure 5E), this was not true for other Nef functions, indicating that B*57 expression alone does not guarantee Nef attenuation in this group.

Rather, our results are consistent with functional variability of naturally occurring Nef sequences from EC, which may be attributable in part to non-canonical HLA-associated escape mutations selected in this rare group. Previously, in order to investigate the influence of HLA-associated viral polymorphisms on HIV-1 protein function in EC, we have made use of reference lists of common HLA-associated polymorphisms derived from population-level studies of chronically infected individuals [20,29]. However, such lists may not capture rare escape mutations that are unique to EC [26,27]. Therefore, we used our EC dataset to identify HLA-associated polymorphisms specific to this population, in a novel analysis of this type. It is important to note that this analysis was aimed at identifying HLA-B*57-associated polymorphisms in the EC dataset itself (and is therefore distinct from the comparative analyses described in [21] that sought to identify polymorphisms enriched among EC compared to CP). Statistical power is a major limitation of this analysis, but HLA-associated polymorphisms can be identified in modestly sized datasets (e.g. [37]), especially if analyses are limited to specific alleles. At p < 0.05, we identified nine B*57-associated polymorphic sites specific to EC (Figure 6A), most of which differed from B*57-associated polymorphisms commonly identified in population-level analyses [20,29]. In contrast, B*57-associated polymorphisms identified in our CP dataset using the same approach were largely as expected [20,29,30]. ELISpot reactivity to overlapping peptides spanning codons 28, 55, 85, 178 and 198 has been documented in B*57+ EC [27]; and V85L has been described to function as an escape mutation in the B*57/58-KF9 epitope [38], further suggesting that these polymorphism may be due to CTL selection pressure in B*57 EC. In addition, paired Nef sequences from PBMC and plasma were available for two B*57 EC in the present study. The first exhibited identical amino acids in both compartments at all 7 of the residues putatively associated with HLA-B*57, while the second exhibited different amino acids at three of the seven B*57-associated sites (codons 85, 105 and 198). Although it is not possible to make conclusions based on only two patients, these data support evolution of these sites *in vivo*.

Notably, we observed dramatic inverse associations between the number of EC-specific B*57-associated polymorphisms and Nef-mediated replication, HLA-I down-regulation and CD74 up-regulation. CD4 down-regulation, the only function in which host expression of B*57 was in itself significantly associated with poorer function in EC, also displayed a modest, albeit not significant, negative relationship between the burden of B*57-associated escape mutations and function (Figure 6). These remarkable inverse relationships were particular to B*57 and not observed for other HLA alleles with similar frequency in our EC cohort. Taken together, results suggest that HLA-B*57-associated CTL pressures select for non-canonical polymorphisms in EC, which contribute additively to multiple functional impairments in EC Nef. Building upon previous studies of recombinant viruses encoding *gag* and *pol* sequences from the same EC cohort [3,4], our results support a complex relationship between B*57-associated immune pressures and Nef function.

In an exploratory analysis of Nef amino acid sequences, we identified 23 polymorphisms, located at 14 residues, associated with Nef function in EC. None overlapped with mutations previously identified in HIV non-progressors [39] or with sites reported to affect HLA-I down-regulation activity in chronic infection [40], a discrepancy that might be due to non-canonical polymorphisms observed in EC. Of interest, 8S was associated with Nef-mediated modulation of cell-surface CD4, HLA-I and CD74, an observation consistent with codon 8’s involvement in myristylation [41]. Also, 28D and 105X (in this case R), associated with modulation of CD74 and CD4, respectively, are EC-specific B*57-associated polymorphisms identified in the present study.

Viral genetic studies of EC feature numerous challenges and limitations. Although care was taken to choose a Nef clone that reflected each patient’s original bulk HIV RNA sequence, and to rule out proviral DNA contamination, potential biases associated with PCR amplification from extremely low copy-number templates must be acknowledged. On the other hand, use of a single sequence per patient alleviates potential biases associated with quasispecies approaches to compare samples with low vs. high genetic diversity. As our goal was to specifically investigate the function of Nef in EC, we employed recombinant virus (and single-protein expression) approaches to eliminate potential confounding effects of other HIV-1 proteins; however, such approaches may not reflect the characteristics of infectious molecular clones or whole-virus isolates recovered from PBMC, procedures that are rarely successful in EC [21,42]. Recombinant virus approaches are also inherently limited by potential incompatibilities between insert and backbone; our choice of a recombinant control strain (NL4.3-Nef_SF2_) alleviates this to a minor extent. Although we assessed Nef activity using primary PBMCs and immortalized cell lines, Nef’s multiple functions [16-18,43] may vary in different cell types [43-45]. Furthermore, the *in vivo* relevance of our observations - in particular, the extent to which these functional differences contribute to the viremia control in EC - remains unclear. Although our results are consistent with the transmission of partially attenuated Nef sequences in at least some EC, and/or further immune-mediated attenuation in others [3,4,46], it is not possible to disentangle cause and effect in cross-sectional studies. Furthermore, Nef function may change over the infection course [47], therefore longitudinal analysis of Nef function in controllers, beginning in the acute/early phase of infection, is warranted. Finally, although our results suggest that non-canonical polymorphisms in EC may contribute to attenuated Nef function, it will be important to validate these findings in larger EC cohorts. Despite these limitations, our study represents the largest linked analysis of multiple *in vitro* Nef functions in EC to date, and to our knowledge the only study assessing CD74 up-regulation and replication capacity in this group.

## Conclusions

EC Nef clones were generally functional; however, all five activities assessed were significantly impaired compared to CP Nef clones. HLA-I-restricted immune pressure, most notably by B*57, may contribute to the differences observed. Taken together with previous studies of HIV Gag, Pol, and Env function in EC [3,4,7], our results support decreased viral protein function as a hallmark of the EC phenotype and underscore the potential role of immune pressures in modulating viral protein function in this rare group.

## Methods

### Study subjects

45 EC (median [interquartile range, IQR] pVL 2 [0.2-14] RNA copies/ml [25]; median [IQR] CD4 count 811 [612–1022] cells/mm^3^) and 46 CP (median [IQR] pVL 80500 [25121–221250] RNA copies/ml); median [IQR] CD4 count 292.5 [72.5-440] cells/mm^3^) were studied as described previously [3,4,21,25]. All EC and CP were HIV-1 subtype B-infected, untreated at the time of sample collection, recruited from the Boston area, and comparable with respect to ethnicity and date of HIV diagnosis (1985–2006 for EC vs. 1981–2003 for CP). This study was approved by the institutional review board of Massachusetts General Hospital, Boston USA; all participants provided written informed consent.

### Cloning and analysis of nef genes

For EC, HIV RNA was extracted from a starting volume of 4.5 to 35.0 ml of plasma and amplified using nested RT-PCR, as described [21]. Given that the median pVL in our EC cohort was 2 RNA copies/ml [IQR 0.2-14] [25], we estimate that on average, 40 viral RNA templates were extracted for each EC patient. To rule out proviral DNA contamination, all extractions included a DNAse treatment step; controls lacking RT enzyme were also performed [21]. For CP, HIV RNA was extracted from 0.5ml of plasma and amplified in the same manner. Nef amplicons were cloned into pIRES2-EGFP expression vector (Clontech). A minimum of three Nef clones were sequenced per patient, and a single clone with an intact Nef reading frame that closely resembled the original bulk plasma RNA sequence was selected. Genbank accession numbers for clonal Nef sequences are JX171199-JX171243 (EC) and JX440926-JX440971 (CP).

### Recombinant virus construction and verification of Nef expression

*Nef* clones were transferred into a pNL4.3ΔNef plasmid as described [23] and confirmed by DNA sequencing. Recombinant viruses harboring *nef* from HIV strain SF2 (NL4.3-Nef_SF2_), and lacking *nef* (NL4.3ΔNef) were used as positive and negative controls, respectively. Infectious viruses were generated as described [48]. Briefly, HEK-293T cells were transfected with each proviral clone. Virus-containing culture supernatants were harvested 48 hr following transfection, titered by p24^Gag^ ELISA (ZeptoMetrix Corp.) and aliquots stored at −80°C until use.

HEK-293T cell pellets collected at this time point were used to prepare total cell lysates as described [45] that were subjected to SDS-PAGE in duplicate and transferred to nitrocellulose membranes. Nef genetic diversity poses a challenge to antibody-based detection as differences in reactivity may reflect suboptimal antibody binding rather than variation in protein levels. To ensure detection of patient-derived Nef, duplicate blots were probed using unique anti-Nef polyclonal antisera, developed from sheep (ARP 444; provided by O.T. Fackler, Heidelberg University, Germany) or rabbit (NIH AIDS Research and Reference Reagent Program). Band intensities were quantified using ImageQuant LAS 4000 (GE Healthcare Life Sciences).

### Virion infectivity and replication assays

Recombinant virus infectivity was determined by exposing 10^4^ TZM-bl cells (NIH AIDS Research and Reference Reagent Program) to 3 ng p24^Gag^ recombinant virus followed by chemiluminescence detection 48 hr later as described [49]. Infectivity values represented the mean of triplicate experiments, normalized to control strain NL4.3-Nef_SF2_, such that values > 100% and < 100% indicated increased or decreased infectivity, respectively. Recombinant virus replication was assessed by exposing 10^6^ freshly isolated PBMC from four HIV-negative donors to 10 ng p24^Gag^ recombinant virus for 8 hr, washing twice, and then resuspending cells in a culture medium (RPMI 1640, 10% FCS) as described [22,23]. Three days later, PBMCs were stimulated with phytohemagglutinin at 5 μg/ml. Culture supernatants were collected and replaced with fresh medium supplemented with human rIL-2 every 3 days. Viral replication was monitored by measuring p24^Gag^ in the culture supernatant using ELISA over 12 days. ELISA values during the initial burst of viral replication (on day 9) were used as our measure of replication capacity. Results were expressed as the mean of quadruplicate assessments in each donor, normalized to control strain NL4.3-Nef_SF2_.

### Analysis of receptor modulation HIV-infected cell surface

To assess Nef-mediated HLA-I down-regulation and CD74 up-regulation, 721.221 cells stably expressing CD4 and HLA-A*24:02 (provided by Masafumi Takiguchi,Kumamoto University, Japan) were exposed to 300 ng p24^Gag^ recombinant virus for 48 hr, followed by staining with anti-HLA-A24-PE (MBL), anti-CD74-Alexa Fluor-647 (BioLegend), 7-amino-actinomycin D (BioLegend), and anti-p24 Gag-FITC (Beckman Coulter), as described [23]. Mean fluorescence intensity (MFI) of each receptor in live p24^Gag^ positive and negative subsets was determined by flow cytometry (FACS Canto II; BD Biosciences). Results were expressed as the mean of duplicate experiments, normalized to control strain NL4.3-Nef_SF2_.

### Analysis of Nef-mediated CD4 down-regulation

To assess Nef-mediated CD4 down-regulation, 3 × 10^5^ CEM-SS cells were transfected with 5 µg plasmid DNA encoding Nef protein and GFP by electroporation (BioRad GenePulser MX) and stained 24 hr later with anti-CD4-APC (BD Biosciences). MFI of GFP-negative and GFP-positive (Nef-expressing) subsets was determined by flow cytometry (Guava easyCyte 8HT, Millipore). Results were normalized to plasmid expressing Nef_SF2_.

### Statistical analyses, including identification of B*57-associated polymorphisms in patient-derived Nef sequences

Phylogenetically-informed methods were used to identify Nef amino acids significantly associated with HLA-B*57allele expression in our EC ( [28], implemented at http://research.microsoft.com/en-us/um/redmond/projects/mscompbio/phylododdsratio/default.aspx). Multiplecomparisons were addressed using q-values, the p-value analogue of the false discovery rate (FDR) [50]. The FDR is the expected proportion of false positives among results deemed significant at a given p-value threshold; for example, at a q ≤ 0.2, we expect 20% of identified associations to be false positives.

## Competing interests

The authors declare that they have no competing interests.

## Authors’ contributions

PM, TJM, ZLB, MAB, and TU designed the study; PM, TJM, EM, YO, XTK, MT, and MM performed the experiments; FP, TM and BDW provided access to patient samples and analyzed clinical data; PM, TJM, EM, ZLB, MAB, and TU analyzed data; and PM, ZLB, MAB, and TU wrote the paper. All authors read and approved the final manuscript.

## Supplementary Material

Additional file 1 **Table S1.** EC Nef activity with aligned amino acid sequence. Click here for file
